# High-Efficiency and Fast Hydrogen Production from Sodium Borohydride: The Role of Adipic Acid in Hydrolysis, Methanolysis and Ethanolysis Reactions

**DOI:** 10.3390/molecules29204893

**Published:** 2024-10-16

**Authors:** Savas Gurdal

**Affiliations:** Science and Technology Research and Application Center, Canakkale Onsekiz Mart University, 17020 Canakkale, Turkey; savas.gurdal@comu.edu.tr

**Keywords:** adipic acid, sodium borohydride, ethanolysis, methanolysis, hydrogen production

## Abstract

In this study, hydrogen production through the hydrolysis, ethanolysis, and methanolysis reactions of NaBH_4_ using adipic acid as a catalyst was investigated for the first time. Adipic acid solutions were prepared with methanol and ethanol at concentrations of 0.1, 0.2, 0.3, 0.4, and 0.5 M. In these reactions, NaBH_4_-MR (methanolysis) and NaBH_4_-ER (ethanolysis) reactions were carried out at 30, 40, and 50 °C with NaBH_4_ concentrations of 1.25%, 2.5%, and 5%. Hydrolysis reactions (NaBH_4_-HR) were conducted at 0.1 M under the same conditions. In the ethanolysis and methanolysis reactions at 30 °C, total hydrogen conversion was achieved at 0.3 M, 0.4 M, and 0.5 M. However, in the hydrolysis reactions, total hydrogen production was only obtained at 50 °C. It was observed that in the NaBH_4_-MR and NaBH_4_-ER reactions, total hydrogen conversion could be achieved within 4–5 s. The utilization of adipic acid as a catalyst for hydrogen production from NaBH_4_ through ethanolysis and methanolysis reactions is proposed as a highly efficient and fast method, characterized by impressive conversion rates.

## 1. Introduction

The escalating energy demands of contemporary society have led to the rapid depletion of fossil fuel reserves and a significant rise in atmospheric CO_2_ emissions. Transitioning from nonrenewable resources, such as fossil fuels, to renewable energy sources is widely seen as a viable solution to mitigate the energy crisis. This shift aims to reduce our reliance on carbon-intensive fossil fuels, thereby promoting a more sustainable and environmentally friendly future [1]. The significance of it lies in the fact that it can produce absolutely no carbon emissions at the point of use and can therefore resolve many environmental problems, including global warming and pollution [2]. Hydrogen is recognized as a novel, clean, and highly efficient energy source, poised to play a crucial role in the future of sustainable fuel. It has garnered sustained research interest and substantial investment due to its potential as a key clean energy solution [3]. The development of efficient hydrogen storage and transportation methods further underlines its potential in creating a resilient and adaptable energy infrastructure [4]. Hydrogen, the most abundant element in the universe, holds a pivotal role in the transition towards a sustainable energy future due to its versatile and clean energy potential. As an energy carrier, hydrogen can be efficiently converted between chemical and electrical energy, making it integral to various sectors including transportation, industry, and power generation [5]. Furthermore, hydrogen can be produced from a variety of renewable sources, including water via electrolysis using solar or wind energy, which enhances its role in achieving energy security and independence from fossil fuels [6]. As the world pivots towards sustainable energy solutions, hydrogen’s role as a clean, efficient, and flexible energy vector becomes increasingly indispensable [7]. This necessity drives ongoing research and innovation in hydrogen production, storage, and utilization technologies to overcome existing challenges and fully realize hydrogen’s potential in the global energy landscape [8].

Sodium borohydride (NaBH_4_) has garnered significant attention as a widely used chemical hydride for generating pure hydrogen for fuel cells. Its high theoretical hydrogen capacity (10.8 wt.%), excellent storability and stability, non-toxicity, safe reaction conditions, and production of environmentally benign byproducts contribute to its appeal [3]. The hydrolysis of sodium borohydride (NaBH_4_), typically catalyzed by various metals or metal composites, efficiently produces hydrogen in aqueous solutions. This reaction offers a controlled and safe method for hydrogen release, which is essential for applications in portable and stationary power sources. Catalysts such as ruthenium nanoclusters and cobalt-based compounds have shown high catalytic activity and stability, significantly enhancing the rate of hydrogen production [9,10]. The successful generation of hydrogen via NaBH_4_ hydrolysis critically depends on the use of suitable catalysts. Recently, significant efforts have been focused on exploring non-noble metal catalysts to improve hydrogen production efficiency from NaBH_4_ solutions [11]. Likewise, the alcoholysis of NaBH_4_, using alcohols such as methanol or ethanol, is a viable alternative, particularly in conditions where water freezing is problematic. This method is advantageous due to fewer steps being required for recycling spent borohydride and the elimination of water-related freezing issues [12,13]. Furthermore, the use of environmentally benign catalysts, such as acetic acid, has proven effective in both hydrolysis and alcoholysis reactions, offering a greener approach to hydrogen production [14,15]. The swift hydrogen generation facilitated by these catalytic processes underscores the promise of sodium borohydride as an eco-friendly and effective hydrogen provider for fuel cell uses.

The commercialization of hydrogen production from NaBH_4_ faces significant challenges, such as limited hydrogen yield at ambient temperature and the inadequate solubility of NaBH_4_ and NaBO_2_ byproducts in highly alkaline solutions. To address these issues, the development of suitable catalysts is essential for enhancing the efficiency of NaBH_4_ hydrolysis reactions [16]. A major issue is the slow reaction rate of NaBH_4_ hydrolysis at room temperature, resulting in an overall conversion rate of only 7–8% [17,18].

Equations (1)–(3) illustrate the hydrolysis (HR), ethanolysis (ER), and methanolysis reactions (MR) of NaBH_4_ in the presence of a catalyst (cat.) [18]:

(1) $${\mathrm{NaBH}}_{4}+4H_{2}O\;\underset{\to }{\mathrm{cat.}}\;{\mathrm{NaBO}}_{2}+4H_{2}$$

(2) $${\mathrm{NaBH}}_{4}+4{\mathrm{CH}}_{3}{\mathrm{CH}}_{2}\mathrm{OH}\;\underset{\to }{\mathrm{cat.}}\;\mathrm{NaB}{\left({\mathrm{OCH}}_{2}{\mathrm{CH}}_{3}\right)}_{4}+4H_{2}$$

(3) $${\mathrm{NaBH}}_{4}+4{\mathrm{CH}}_{3}\mathrm{OH}\;\underset{\to }{\mathrm{cat.}}\;\mathrm{NaB}{\left({\mathrm{OCH}}_{3}\right)}_{4}+4H_{2}$$

The methanolysis reaction of sodium borohydride occurs spontaneously. Previous studies indicate that the kinetic reaction constant for the spontaneous alcoholysis of sodium borohydride is higher than that of its spontaneous hydrolysis at ambient temperature without the addition of any catalyst [19]. Numerous catalysts have been investigated and developed for the production of H_2_ from the methanolysis and hydrolysis of NaBH_4_ [20,21]. The acid hydrolysis of sodium borohydride (NaBH_4_) typically involves the gradual addition of an aqueous acid solution to solid NaBH_4_ powder. This method has several advantages, including the production of dry hydrogen gas, ease of hydrogen generation control, and environmentally friendly waste byproducts resulting from the reaction [22]. Acid solutions offer the added benefit of being easily stored as liquids at various concentrations, while efficiently consuming the available hydrogen ions in the solution [14]. However, one of the primary challenges of NaBH_4_ hydrolysis is the slow reaction rate at room temperature. As a result, effective catalysts, whether metal or non-metal, are necessary to reduce the activation energy and enhance the hydrogen generation rate [18]. Acid catalysts are particularly advantageous for NaBH_4_ hydrolysis due to their ability to be stored in various concentrations as aqueous solutions. Moreover, they enable the reaction to occur at room temperature, while the byproduct, sodium metaborate, is less toxic and environmentally benign [23]. Boric acid and its derivatives have also been highlighted as green catalysts in various acid-catalyzed reactions, contributing to the environmentally friendly nature of the process [24]. Schlesinger et al. [25] investigated various acid catalysts for hydrogen production and demonstrated that the complete hydrolysis of NaBH_4_ can be achieved rapidly with the addition of acid. Typically, acid hydrolysis of NaBH_4_ is performed by gradually adding an aqueous acid solution to solid NaBH_4_ powder. This method’s primary advantages include the production of very dry hydrogen gas, easy control over hydrogen production, and the environmentally benign nature of the waste products formed during the reaction [26]. In line with this, Yuanyu Xia and colleagues systematically investigated the catalytic performance of CoB catalysts doped with various elements such as Sn, Mn, Cr, and Bi. These dopants were selected to enhance the activity of the CoB catalyst, and the study compared the phase structure, surface morphologies, electronic interactions, and specific surface areas of these ternary alloy catalysts. Their research demonstrated significant improvements in hydrogen production from NaBH_4_, highlighting the potential of dopant-enhanced CoB catalysts [27]. In the past, the NaBH_4_ hydrogen production system initially utilized catalysts such as oxalic, boric, formic, and citric acids. The findings revealed that these acids significantly influenced the NaBH_4_ reaction, prompting a search for safer alternatives [23]. Typically, a range of acids, including phosphoric acid, nitric acid, acetic acid, sulfuric acid, hydrochloric acid, and formic acid, are employed in the hydrolysis reactions of NaBH_4_ [20]. Furthermore, acetic acid (CH_3_COOH), an eco-friendly weak organic acid that can be derived from bio-based processes, was explored as a catalyst promoter. In that study, acetic acid was used to acidify the alkaline NaBH_4_ solution, effectively promoting the hydrolysis reaction. Beyond its environmental advantages, acetic acid facilitated NaBH_4_ hydrolysis with operational simplicity and strong catalytic performance, offering a practical solution for efficient hydrogen production [28]. Utilizing acid catalysts in the hydrolysis reaction of NaBH_4_ presents multiple benefits: these catalysts can be maintained in various concentrations as aqueous solutions, the reactions can take place at ambient temperature, and the sodium meta-borate byproduct is relatively non-toxic and eco-friendly [29]. Expanding on the search for efficient catalysts, Dijit and co-workers reported the use of hydrothermally synthesized cobalt sulfide (CoS) nanoparticles for NaBH_4_ hydrolysis. The porous and polycrystalline nature of the CoS nanoparticles accelerated hydrogen generation, achieving a rate of 328 mL min^−1^ g^−1^ at room temperature (25 °C) with a 2.9% NaBH_4_ solution. This rate increased to 551 mL min^−1^ g^−1^ at 50 °C with a 1% NaBH_4_ solution, demonstrating the enhanced catalytic effect at higher temperatures [30].

Methanol and ethanol present several advantages compared to water in alcoholysis reactions. Firstly, the reaction kinetics of NaBH_4_ or KBH_4_ with these alcohols are considerably faster. Secondly, their lower melting points lead to improved catalytic performance at reduced temperatures. The alcoholysis reactions do not generate undesirable byproducts, enhancing the overall efficiency and cleanliness of the process. In contrast to the sticky and hydrated byproduct produced during the hydrolysis reaction, the byproduct of the methanolysis reaction, NaB(OCH_3_)_2_, does not exhibit a tendency to clog the reactor. Additionally, methanol’s low freezing point presents a significant advantage for hydrogen generation in subzero temperatures, where the water required for hydrolysis would otherwise freeze and hinder the reaction. Although the theoretical hydrogen storage capacity of a methanolysis-based system is limited to only 4.9 wt.%, the methanolysis of sodium borohydride holds potential for efficient hydrogen production at low temperatures, making it a promising alternative under such conditions [21,24].

Adipic acid, also known as hexanedioic acid, is an organic compound with the chemical formula (CH_2_)_4_(COOH)_2_. Industrially, it is the most significant dicarboxylic acid, with an annual production of approximately 2.5 billion kilograms, primarily used as a precursor in nylon manufacturing, and adipic acid is rarely found in nature in its natural form [31]. Adipic acid, with a molecular mass of 146.14 g mol^−1^ and pKa values of 4.43 and 5.41, is one of the most commercially significant aliphatic, straight-chain dicarboxylic acids [32]. It is primarily utilized in the production of nylon 6-6, and adipic acid catalysts are rarely employed in alcoholysis reactions. Adipic acid, used in some reactions, is known for its biodegradability and low acute toxicity, further supporting its use in sustainable chemistry [33].

Balbay, A. & Saka, C. investigated the concentration impacts of NaBH_4_, along with the concentrations of hydrochloric acid and acetic acid, and temperature on the reactions. The highest hydrogen production rates in semi-methanolysis reactions using 1 M hydrochloric acid and acetic acid were 4875 mL min^−1^ and 3960 mL min^−1^, respectively. These semi-methanolysis reactions with the acids were completed within 4 and 5 s, respectively [34]. In another study of Balbay, A. & Saka, C., the catalytic activity of the catalysts was evaluated by measuring the hydrogen production rate during the acidified hydrolysis of NaBH_4_. The highest hydrogen production rates in the hydrolysis reaction with 0.25 M H_3_PO_4_, using a Cu-based catalyst prepared in water and methanol solvents, were 825 mL g^−1^ min^−1^ and 660 mL g^−1^ min^−1^, respectively [26]. Dandan Ke et al. studies about the hydrolysis reaction catalyzed by a Mo-modified Co-B catalyst achieves a maximum hydrogen generation rate of 4200 mL H_2_ min^−1^ g^−1^ catalyst and an activation energy of 43.7 kJ mol^−1^, surpassing the performance of the Co-B catalyst. Kinetic studies indicate that at low NaBH_4_ concentrations, the reaction follows first-order kinetics with respect to NaBH_4_ concentration, suggesting that the rate-limiting step is the surface adsorption of BH_4_^−^ [35]. Arzac, G. M. & Fernández, A. studied some acids for hydrogen generation experiments. Acetic acid has demonstrated the optimal balance between hydrogen generation rates, conversion efficiency (which increases with the amount of accelerator) and environmental friendliness. The highest experimental gravimetric hydrogen density (GHD) achieved was 2.1 wt.%. Adding water to ethanol increases hydrogen production rates but decreases conversion efficiency. Additionally, utilizing ethanol–methanol mixtures without any catalyst reduces hydrogen production rates. Arzac and Fernandez investigated the NaBH_4_ ethanolysis process using acetic acid as a catalyst, varying the amount of acetic acid accelerator from 3 to 9 mg per 1 mL of ethanol. Their results demonstrated that the hydrogen production rate (r50) increased as the mass of the catalyst increased. The activity of the reaction was calculated as 2800 mL min^−1^ g^−1^ based on the slope, and it was observed that the conversion efficiency improved with the increasing amount of acetic acid. The study also examined the performance of different acidic accelerators in NaBH_4_ ethanolysis. Among them, HCl exhibited the highest reaction rate of 5020 mL min^−1^ g^−1^ with a total conversion of 77%. Acetic acid, although yielding a lower reaction rate of 2000 mL min^−1^ g^−1^, achieved the highest conversion rate of 90%. Citric acid showed a reaction rate of 460 mL min^−1^ g^−1^ with 80% conversion, while phthalic acid had a slightly lower reaction rate of 400 mL min^−1^ g^−1^ and 71% conversion. Both boric and tartaric acids demonstrated similar performances, each with a reaction rate of 360 mL min^−1^ g^−1^ and a conversion efficiency of 72%. In contrast, ascorbic acid resulted in a significantly lower conversion efficiency of 43%, with no hydrogen production rate (r50) reported. These findings indicate that acetic acid serves as an effective catalyst in NaBH_4_ ethanolysis, offering a high conversion rate despite a moderate reaction rate. However, HCl stands out for its superior reaction rate, though its conversion efficiency does not exceed that of acetic acid. The comparison of these acidic accelerators suggests that each has distinct advantages depending on the desired balance between reaction speed and overall conversion efficiency [15]. Wang and colleagues introduced an innovative RueNi/Ni foam catalyst that demonstrated high efficiency for NaBH_4_ methanolysis. This catalyst was developed using a combination of electroless plating and electroplating techniques, achieving a hydrogen generation rate of 360 mL min^−1^ g^−1^. Their work highlights the potential of RueNi/Ni foam as an effective catalyst for hydrogen production [36]. In another study, Saka & Balbay investigated hydrogen evolution from the alcoholysis of sodium borohydride using H_3_BO_3_ as a catalyst. Varying concentrations of H_3_BO_3_ (0.2, 0.4, 0.5, 1 M, and a saturated solution) resulted in completion times for the NaBH_4_-methanolysis reaction of 50, 15, 10, 2, and 1 min, respectively. These results demonstrate the significant influence of H_3_BO_3_ concentration on the reaction rate and its potential as an effective catalyst for rapid hydrogen production [13]. In another recent study by Kotkondawar and Rayalu, a ternary Co-Ce-Pt nanocomposite, immobilized on carbon-grafted graphene oxide, was synthesized through a one-step co-reduction process. This nanocomposite exhibited an exceptional hydrogen generation rate, suggesting that advanced nanocomposite structures can significantly enhance catalytic performance [37]. Additionally, Co-Mo-P/CNTs-Ni foam has been shown to effectively catalyze the alcoholysis of sodium borohydride, producing hydrogen at a rate of 2.64 L min^−1^ g^−1^. This reaction displayed a notably lower activation energy of 47.27 kJ mol^−1^, far lower than the spontaneous alcoholysis reaction of NaBH_4_, underscoring the role of Co-Mo-P/CNTs in facilitating hydrogen production at reduced energy costs [38]. Furthermore, Ni-CoB hollow nanospheres, synthesized within 60 min (denoted as NieCoe B-60), demonstrated superior catalytic activity for NaBH_4_ hydrolysis at 303 K. These nanospheres achieved a hydrogen generation rate of 6400 mL min^−1^ g^−1^ and an activation energy of 33.1 kJ mol^−1^, establishing them as highly effective catalysts for efficient hydrogen production [39]. In a related study, Alshammari and colleagues aimed to develop a cost-effective and efficient nanocomposite catalyst for hydrogen production. Using a simple synthesis method, they prepared a Cr_2_O_3_-doped CaCO_3_ nanocomposite, which served as a catalyst for the methanolysis of NaBH_4_. This new nanocomposite successfully promoted hydrogen generation, adding to the growing list of innovative catalyst materials designed for efficient hydrogen production from NaBH_4_ [40]. Together, these studies illustrate the ongoing advancements in catalyst design for NaBH_4_ methanolysis and alcoholysis, each demonstrating unique catalytic efficiencies and mechanisms that contribute to the broader goal of sustainable hydrogen production.

The purpose of this study is to evaluate the effectiveness of adipic acid as a catalyst in promoting hydrogen production through the hydrolysis, methanolysis, and ethanolysis reactions of sodium borohydride (NaBH_4_). While extensive research has been conducted on NaBH_4_ reactions in water, limited studies have focused on its reactivity in methanol and ethanol, and the use of adipic acid as a catalyst in these alcoholysis and hydrolysis processes has rarely been explored. This research aims to investigate the catalytic role of adipic acid in these reactions, specifically assessing its ability to accelerate hydrogen generation and improve conversion rates in each medium.

## 2. Result and Discussion

**Figure 1:**
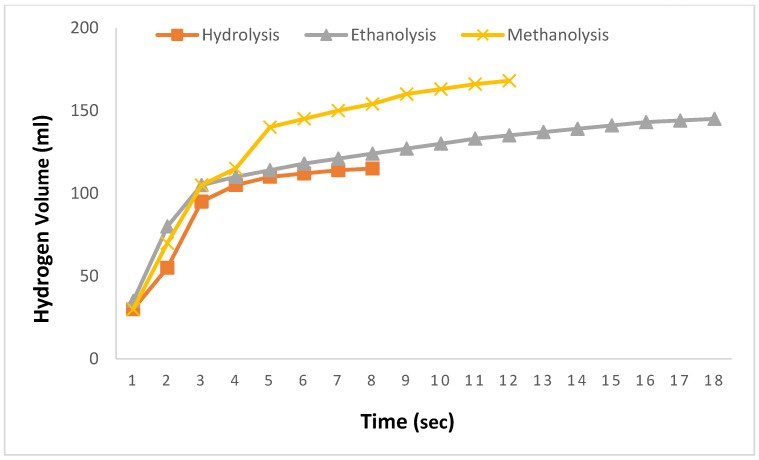
Hydrogen generation quantities from hydrolysis, methanolysis, and ethanolysis reactions of 2.5% NaBH_4_ in the presence of a 0.1 M adipic acid catalyst at 30 °C.

In Figure 1, the results of hydrolysis, methanolysis, and ethanolysis reactions using prepared 0.1 M aqueous, methanol, and ethanol solutions of adipic acid at 30 °C with a 2.5% NaBH_4_ solution are presented. The comparison is limited to 0.1 M solutions because adipic acid can only be dissolved up to 0.1 M in water before reaching saturation. Upon comparing the results in Figure 1, the theoretical expectation of hydrogen production for 2.5% NaBH_4_ was not achieved. In the hydrolysis reaction, hydrogen production reached 115 mL at the 8th second, after which no further hydrogen production was observed despite extended waiting periods. During the ethanolysis reaction, 145 mL of hydrogen was produced in 18 s, whereas the methanolysis reaction yielded 168 mL of hydrogen within a short period of 12 s, with no further production observed afterward despite prolonged waiting. As illustrated in Figure 1, the 0.1 M methanolysis reaction facilitated faster and greater hydrogen production compared to the other reactions.

**Figure 2:**
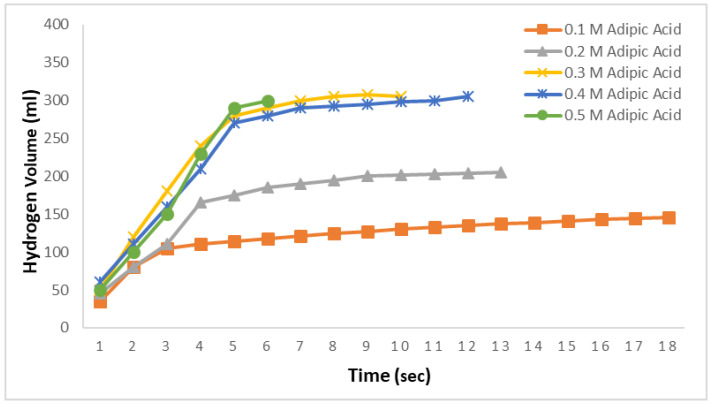
Impact of adipic acid concentration on hydrogen generation in ethanolysis reactions with 2.5% NaBH_4_ at 30 °C.

In Figure 2, the hydrogen production amounts from 2.5% NaBH_4_ in 0.1 M, 0.2 M, 0.3 M, 0.4 M, and 0.5 M adipic acid ethanol solutions at 30 °C are examined. The objective here is to investigate the effects of adipic acid concentrations on ethanolysis reactions. As observed in Figure 2, the theoretical hydrogen production was not achieved for the 0.1 M and 0.2 M solutions. However, with increasing adipic acid concentration, it was found that all the hydrogen contained in NaBH_4_ was released in a short period. These times were observed to be 10 s for the 0.3 M solution, 12 s for the 0.4 M solution, and very rapidly at 6 s for the 0.5 M solution.

**Figure 3:**
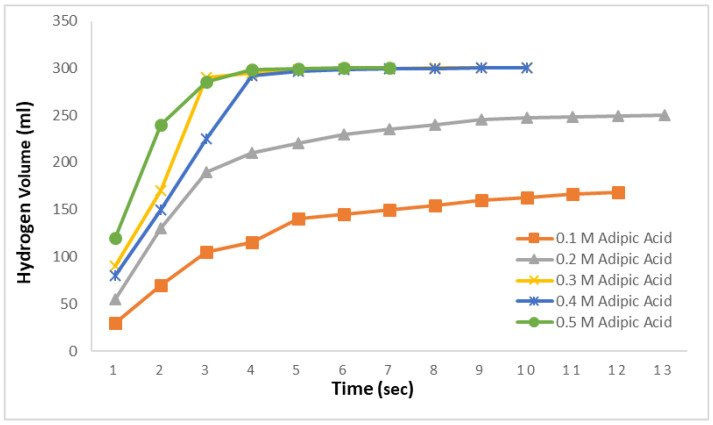
Impact of adipic acid concentration on hydrogen generation in methanolysis reactions with 2.5% NaBH_4_ at 30 °C.

Figure 3 replicates the study conducted in Figure 2, this time for methanolysis reactions. In this context, reactions were carried out using a 0.1–0.5 M adipic acid methanol solutions at 30 °C with 2.5% NaBH_4_. The results indicated that hydrogen production was rapid, with 168 mL produced in 12 s for the 0.1 M solution and 250 mL in 13 s for the 0.2 M solution. Beyond these times, no further hydrogen production was observed despite prolonged observation. In the methanolysis reactions for the 0.3 M, 0.4 M, and 0.5 M solutions, the theoretically expected hydrogen amount was obtained very quickly. The total hydrogen content in 2.5% NaBH_4_ was completely released within 10, 10, and 7 s, respectively. Remarkably, in the 0.5 M solution, 99% of the hydrogen was produced in just 4 s, with the remaining 1% generated in the next 3 s. This demonstrates that nearly all the hydrogen in the 0.5 M adipic acid methanolysis reaction can be produced very rapidly, within approximately 4 s.

**Figure 4:**
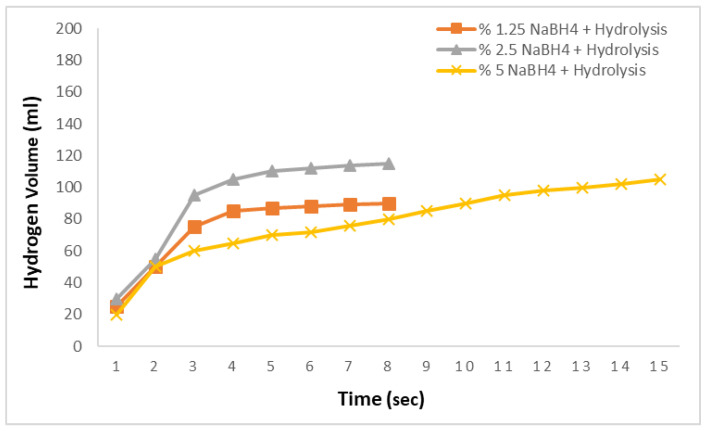
Impact of the NaBH_4_ concentration on the hydrogen generation in the NaBH_4_-HR with a 0.1 M adipic acid concentration at 30 °C.

In the subsequent studies, the amounts of NaBH_4_ were varied, and aqueous solutions at concentrations of 1.25%, 2.5%, and 5% were prepared. Reactions were conducted with these solutions at 0.1 M and 30 °C to examine the hydrogen production rate under the same conditions with varying NaBH_4_ concentrations. Figure 4 presents the results of the hydrolysis reactions using adipic acid. For the 1.25% and 2.5% NaBH_4_ solutions, the hydrogen production rates were similar, yielding 90 mL and 115 mL of hydrogen in 8 s, respectively, with no further production observed thereafter. For the 5% NaBH_4_ solution, however, the hydrogen production rate was significantly slower, producing 105 mL of hydrogen in 15 s. This indicates that the hydrolysis reactions using adipic acid exhibit very slow hydrogen production rates.

**Figure 5:**
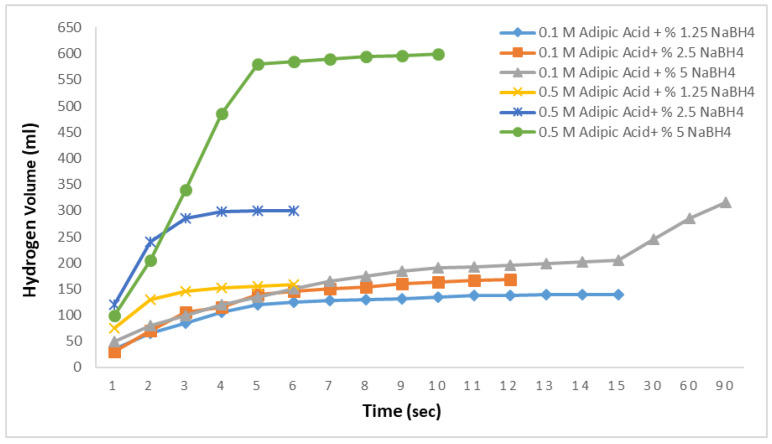
Impact of the NaBH_4_ concentration on the hydrogen generation in the NaBH_4_-MR with 0.1 M and 0.5 M adipic concentrations at 30 °C.

To produce the results displayed in Figure 5, solutions of NaBH_4_ at concentrations of 1.25%, 2.5%, and 5% were prepared, and methanolysis reactions were conducted at 30 °C using adipic acid concentrations of 0.1 M and 0.5 M. The objective here is to examine the hydrogen production rates of different concentrations of NaBH_4_ under varying adipic acid concentrations. At 0.1 M, 140 mL of hydrogen was obtained in 15 s from the 1.25% NaBH_4_ solution. For the 2.5% solution, the hydrogen production rate slowed down, yielding 168 mL in 12 s, and the expected amount of hydrogen was not achieved. In the 0.1 M 5% solution, nearly half of the theoretically expected hydrogen was obtained in 90 s, with no further hydrogen production observed. In the methanolysis reactions with 0.5 M adipic acid, the theoretically expected hydrogen was fully produced in 6 s for both the 1.25% and 2.5% NaBH_4_ solutions. For the 5% NaBH_4_ solution, 98% of the hydrogen was produced in 6 s, with the remainder produced within a total of 10 s. These results demonstrate that increasing the concentration of adipic acid in methanolysis reactions enables very rapid and efficient hydrogen production. Moreover, even with an increase in NaBH_4_ concentration, there was no significant decrease in the hydrogen production rate.

**Figure 6:**
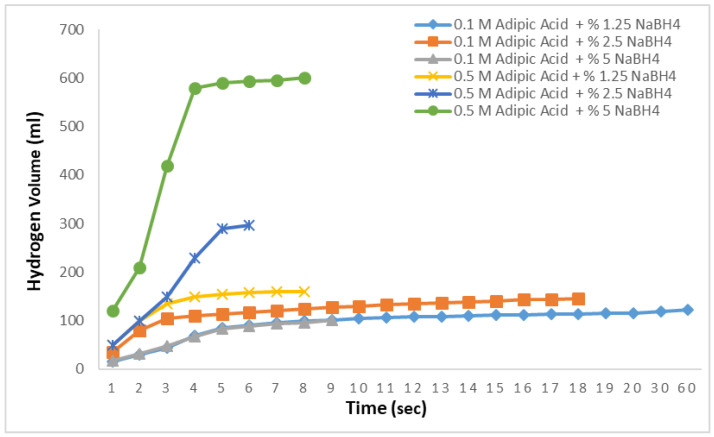
Impact of the NaBH_4_ concentration on the hydrogen generation in the NaBH_4_-ER with a 0.1 M and 0.5 M adipic concentration at 30 °C.

To produce the results shown in Figure 6, as in Figure 5, NaBH_4_ solutions at concentrations of 1.25%, 2.5%, and 5% were prepared, and ethanolysis reactions were conducted at 30 °C using adipic acid concentrations of 0.1 M and 0.5 M. The results of these reactions indicated that hydrogen production was low for all three NaBH_4_ concentrations at 0.1 M, and the expected hydrogen yield was not achieved despite prolonged waiting periods. At a 0.5 M adipic acid concentration, all the hydrogen was produced in 8 s for the 2.5% NaBH_4_ solution, while for the 1.25% solution, the total hydrogen yield was achieved in 6 s. For the 5% NaBH_4_ solution, 99% of the hydrogen was produced in 6 s, with the remaining hydrogen obtained within 8 s.

**Figure 7:**
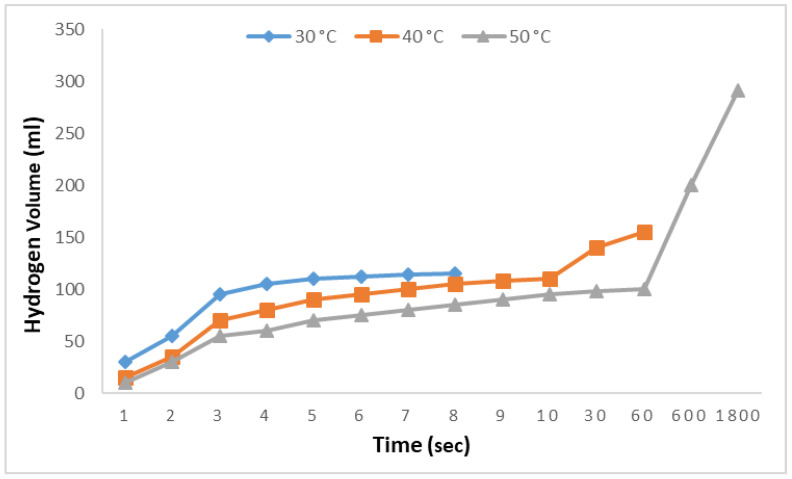
Impact of temperature on hydrogen generation in NaBH_4_-HR with a 0.1 M adipic acid concentration and 2.5% NaBH_4_.

Finally, to produce the results shown in Figure 7, the effects of temperature variations on the rates of hydrolysis and alcoholysis reactions were investigated. In this phase, 0.1 M aqueous adipic acid solutions were examined at 30 °C, 40 °C, and 50 °C with 2.5% NaBH_4_ solutions. At 30 °C, 115 mL of hydrogen was produced in 8 s, followed by an observation period of 1800 s during which no further hydrogen production was detected. At 40 °C, an increase in both the rate and amount of hydrogen production was observed, with 152 mL of hydrogen produced in 50 s; however, no additional hydrogen was produced during the subsequent 1800 s. At 50 °C, although the initial rate was slow, it gradually increased, and nearly all the hydrogen was produced within 1800 s. These results indicate that increasing the temperature has a significant effect on the hydrolysis reactions of adipic acid.

**Figure 8:**
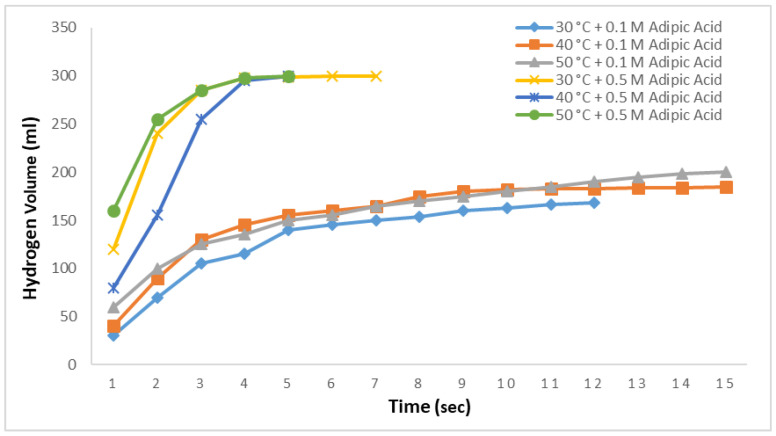
Impact of temperature on hydrogen generation in NaBH_4_-MR with 0.1 M and 0.5 M adipic concentrations and 2.5% NaBH_4_.

As in Figure 7, methanolysis reactions were conducted at three different temperatures using 0.1 M and 0.5 M adipic acid methanol solutions with 2.5% NaBH_4_ aqueous solution and the results are shown in Figure 8. At 0.1 M, 168 mL of hydrogen was produced at 30 °C in 12 s, 185 mL at 40 °C in 14 s, and 200 mL at 50 °C in 14 s. In all three cases, no further hydrogen production was observed during a subsequent 1800 s observation period. At a 0.5 M adipic acid concentration, 99.5% of the hydrogen was produced within 4 s for all three temperatures. Hydrogen production was completed in 7 s at 30 °C and in 5 s at 40 °C and 50 °C.

**Figure 9:**
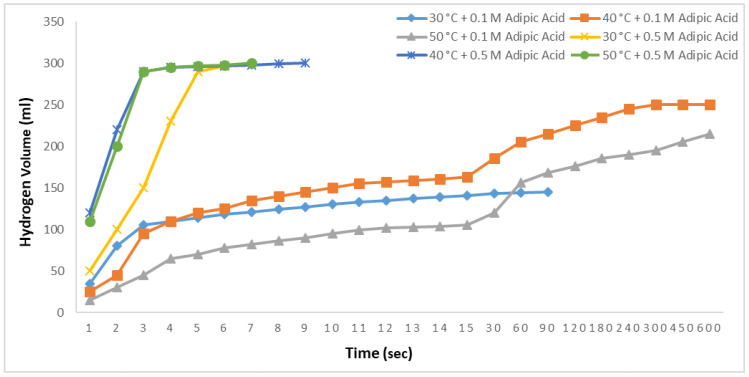
Impact of temperature on hydrogen generation in NaBH_4_-ER with 0.1 M and 0.5 M adipic concentrations and 2.5% NaBH_4_.

Ethanolysis reactions were conducted at 30, 40, and 50 °C with 2.5% NaBH_4_ and adipic acid concentrations of 0.1 M and 0.5 M, and the results are shown in Figure 9. All reactions were observed for up to 600 s. In the 0.1 M studies, at 30 °C, 145 mL of hydrogen was produced in 18 s, with no further hydrogen release observed thereafter. At 40 °C, 186 mL of hydrogen was produced in 30 s, and a total of 200 mL was obtained by 600 s. At 50 °C, 120 mL of hydrogen was produced in 30 s, and a total of 215 mL was obtained by 600 s. The lower hydrogen yield at 50 °C compared to 40 °C could be attributed to the 0.1 M concentration of adipic acid. In the 0.5 M adipic acid studies, all the hydrogen was produced in 6 s at 30 °C. At 40 °C, 99.5% of the hydrogen was produced in 4 s, with total production achieved in 9 s. Finally, at 50 °C, 99.5% of the hydrogen was produced in 4 s, with complete production in 6 s.

Balbay, A. & Saka, C. [13] examined concentration impacts of NaBH_4_ as well as the concentrations of acetic acid and hydrochloric acid achieving high-yield hydrogen production within 4 to 5 s. Similarly, in the present study involving adipic acid, it was found that as the concentration of adipic acid increased, the total hydrogen content of NaBH_4_ could be obtained within 4 to 5 s in both methanolysis and ethanolysis reactions.

## 3. Materials and Methods

Chemicals in the reactions were analytical grade. Adipic acid (≥99.5%, HPLC) was obtained from Sigma (Taufkirchen, Germany). Ethanol (≥99.9%) was provided by Isolab (Eschau, Germany) and methanol (99.8%) by Aldrich(Taufkirchen, Germany). NaBH_4_ (97%) was sourced from AFG Bioscience (Northbrook, IL, USA) for the reactions.

**Figure 10:**
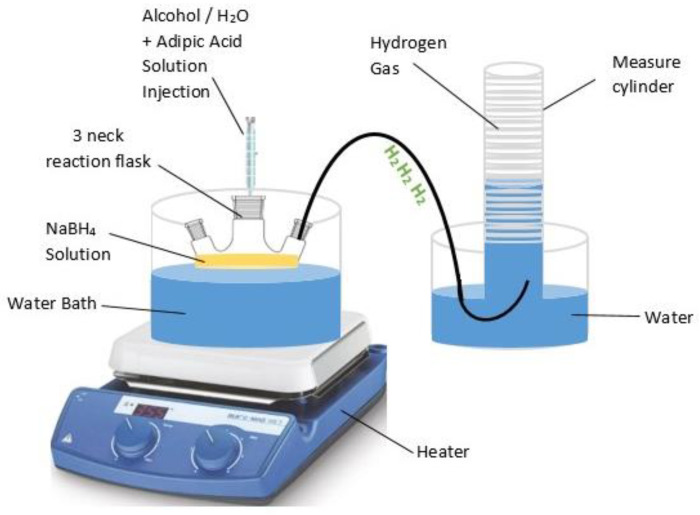
The schematic diagram of the hydrogen production system.

The catalytic efficacy of adipic acid was assessed by measuring the hydrogen yield from NaBH_4_-HR, NaBH_4_-ER, and NaBH_4_-MR. A reaction vessel was immersed in a thermostatically controlled water bath, with temperatures varying between 30 and 50 °C. The hydrogen gas generated was channeled through suitable conduits into an inverted burette for collection and analysis. The reaction vessel was immersed in a thermostatically controlled water bath, with temperatures varying between 30 and 50 °C. One of the necks of the flask was sealed with a cork stopper connected to a hose, allowing the generated hydrogen gas to flow through the hose into a wet gas meter for measurement. The volume of hydrogen produced was recorded using this gas meter. Initially, the two remaining necks of the flask were left open to facilitate the addition of reagents, but they were promptly sealed after the catalyst was introduced into the solution to prevent any gas from escaping. The hydrogen gas generated during the reaction was collected in an inverted burette, which was linked to the system via appropriate tubing for accurate measurement and analysis. To ensure precision, all gas flow pathways were tightly sealed to avoid any potential leaks, and measurements were taken at regular intervals throughout the reaction process to monitor hydrogen production rates. Additionally, care was taken to maintain constant agitation of the reaction mixture to ensure uniform distribution of the catalyst and optimal reaction conditions. Subsequently, 5 mL of NaBH_4_-water solutions at concentrations of 1.25%, 2.5%, and 5% were added to the 50 mL processing flask. Then, 5 mL of adipic acid solutions in methanol, ethanol, or water at concentrations of 0.1, 0.2, 0.3, 0.4, and 0.5 M were introduced into the reaction flask containing NaBH_4_-MR, NaBH_4_-ER, and NaBH_4_-HR. The stoichiometric ratio of adipic acid to NaBH_4_ for the hydrogen generation (HG) reactions was kept constant at 1:1 (*v*/*v*). The volume of hydrogen gas produced over time was quantified using a piston displacement apparatus filled with water. Hydrogen gas measurements were systematically recorded via a high-speed camera. The schematic diagram of the hydrogen production system is shown in Figure 10.

## 4. Conclusions

In this study, hydrogen production through the hydrolysis, ethanolysis, and methanolysis reactions of NaBH_4_ using adipic acid as a catalyst was investigated for the first time. Solutions of adipic acid were prepared with methanol and ethanol at concentrations ranging from 0.1 M to 0.5 M. The NaBH_4_-MR (methanolysis), NaBH_4_-ER (ethanolysis) reactions were carried out at varying NaBH_4_ concentrations and at temperatures of 30, 40, and 50 °C. The hydrolysis reactions (NaBH_4_-HR) were conducted at 0.1 M due to the solubility of adipic acid in water under the same conditions. The volume ratio of adipic acid to NaBH_4_ for the hydrogen generation (HG) reactions was maintained at 1:1 (*v*/*v*). In the ethanolysis and methanolysis reactions at 30 °C, total hydrogen conversion was achieved at 0.3 M, 0.4 M, and 0.5 M. However, in the hydrolysis reactions, total hydrogen conversion was only observed at 50 °C, while total production was not observed at the other temperatures. Additionally, it was found that in the NaBH_4_-MR and NaBH_4_-ER reactions using adipic acid, total hydrogen conversion could be achieved within 4–5 s.

The findings of this study demonstrate that adipic acid is a highly effective catalyst for hydrogen production from NaBH_4_, particularly in methanolysis (MR), ethanolysis (ER), and hydrolysis (HR) reactions. When comparing with the literature, Arzac and Fernandez achieved hydrogen generation rates ranging from 360 to 5020 mL min^−1^ g^−1^ using acidic catalysts like acetic acid and HCl [15]. In contrast, this study achieved complete hydrogen conversion within just 4–5 s via NaBH_4_-MR and NaBH_4_-ER reactions at 30 °C with adipic acid, even at concentrations as low as 0.3 M to 0.5 M. Wang et. al. studied RueNi/Ni foam catalyst demonstrated a hydrogen generation rate of 360 mL min^−1^ g^−1^, significantly slower than the rapid conversion seen in this study [36]. Similarly, Kotkondawar and Rayalu’s [37] Co-Ce-Pt nanocomposite exhibited high hydrogen production but did not achieve such fast reaction times. For hydrolysis studies [38], while Co-Mo-P/CNTs-Ni foam and Ni-CoB nanospheres reached hydrogen production rates of 2.64 L min^−1^ g^−1^ and 6400 mL min^−1^ g^−1^, respectively study demonstrated complete hydrogen conversion only at 50 °C, in line with the temperature dependency observed in the literature. Notably, the results with adipic acid suggest superior catalytic efficiency in alcoholysis reactions compared to phosphoric acid (5779 mL min^−1^ g^−1^) and boric acid studies [26], where completion times ranged from 1 to 50 min depending on the concentration. Overall, adipic acid offers a highly efficient and rapid hydrogen production method, particularly for NaBH_4_ ethanolysis and methanolysis. This represents a novel, rapid, and effective method that will contribute significantly to the literature.

## Data Availability

All the datas were reported in this manuscript.

## References

[1] Zhong H., Iguchi M., Chatterjee M., Himeda Y., Xu Q., Kawanami H. (2018). Formic acid-based liquid organic hydrogen carrier system with heterogeneous catalysts. Adv. Sustain. Syst..

[2] Møller K.T., Jensen T.R., Akiba E., Li H. (2017). Hydrogen—A sustainable energy carrier. Prog. Nat. Sci. Mater. Int..

[3] Saka C., Şahin O., Demir H., Karabulut A., Sarikaya A. (2015). Hydrogen generation from sodium borohydride hydrolysis with a Cu-Co-based catalyst: A kinetic study. Energy Sources Part A Recovery Util. Environ. Eff..

[4] Abdalla A.M., Hossain S., Nisfindy O.B., Azad A.T., Dawood M.M., Azad A.K. (2018). Hydrogen production, storage, transportation and key challenges with applications: A review. Energy Conv. Manag..

[5] Pivovar B., Rustagi N., Satyapal S. (2018). Hydrogen at Scale (H_2_@Scale): Key to a clean, economic, and sustainable energy system. Electrochem. Soc. Interface.

[6] Nishiyama H., Yamada T., Nakabayashi M., Maehara Y., Yamaguchi M., Kuromiya Y., Nagatsuma Y., Tokudome H., Akiyama S., Watanabe T. (2021). Photocatalytic solar hydrogen production from water on a 100-m^2^ scale. Nature.

[7] Balat M., Kırtay E. (2010). Hydrogen from biomass—Present scenario and future prospects. Int. J. Hydrogen Energy.

[8] Sdanghi G., Maranzana G., Celzard A., Fierro V. (2019). Review of the current technologies and performances of hydrogen compression for stationary and automotive applications. Renew. Sustain. Energy Rev..

[9] Özkar S., Zahmakiran M. (2005). Hydrogen generation from hydrolysis of sodium borohydride using Ru(0) nanoclusters as catalyst. J. Alloys Compd..

[10] Wei Y., Wang M., Feng W., Li W., Zhao X., Zhou X., Ni M., Wang H. (2020). Highly active and durable catalyst for hydrogen generation by the NaBH_4_ hydrolysis reaction: CoWB/NF nanodendrite with an acicular array structure. J. Alloys Compd..

[11] Xu F., Ren J., Ma J., Wang Y., Zhang K., Cao Z., Sun Q., Wu S., Li G., Bai S. (2024). A review of hydrogen production kinetics from the hydrolysis of NaBH_4_ solution catalyzed by Co-based catalysts. Int. J. Hydrogen Energy.

[12] Ramya K., Dhathathreyan K.S., Sreenivas J., Kumar S., Narasimhan S. (2013). Hydrogen production by alcoholysis of sodium borohydride. Int. J. Energy Res..

[13] Saka C., Balbay A. (2020). Influence of process parameters on enhanced hydrogen evolution from alcoholysis of sodium borohydride with a boric acid catalyst. Int. J. Hydrogen Energy.

[14] Akdim O., Demirci U., Miele P. (2009). Acetic acid, a relatively green single-use catalyst for hydrogen generation from sodium borohydride. Int. J. Hydrogen Energy.

[15] Arzac G.M., Fernández A. (2015). Hydrogen production through sodium borohydride ethanolysis. Int. J. Hydrogen Energy.

[16] Shang Y., Chen R. (2011). Thermodynamic Study on the Solubility of NaBH_4_ and NaBO_2_ in NaOH Solutions.

[17] Kojima Y., Kawai Y., Nakanishi H., Matsumoto S. (2004). Compressed hydrogen generation using chemical hydride. J. Power Sources.

[18] Demirci U.B., Garin F. (2008). Promoted sulphated-zirconia catalysed hydrolysis of sodium tetrahydroborate. Catal. Commun..

[19] Sahiner N., Yasar A.O., Aktas N. (2017). H_2_ generation from NaBH_4_ methanolysis via magnetic field sensitive ionic liquid coated silica particles as catalyst. Surf. Interfaces.

[20] Lo C.-T.F., Karan K., Davis B.R. (2009). Kinetic assessment of catalysts for the methanolysis of sodium borohydride for hydrogen generation. Ind. Eng. Chem. Res..

[21] Hannauer J., Demirci U.B., Pastor G., Geantet C., Herrmann J.M., Miele P. (2010). Hydrogen release through catalyzed methanolysis of solid sodium borohydride. Energy Environ. Sci..

[22] Brack P., Dann S.E., Wijayantha K.G.U. (2015). Heterogeneous and homogeneous catalysts for hydrogen generation by hydrolysis of aqueous sodium borohydride (NaBH_4_) solutions. Energy Sci. Eng..

[23] Abdul-Majeed W.S., Arslan M.T., Zimmerman W.B. (2014). Application of acidic accelerator for production of pure hydrogen from NaBH_4_. Int. J. Ind. Chem..

[24] Hansen T.S., Mielby J., Riisager A. (2011). Synergy of boric acid and added salts in the catalytic dehydration of hexoses to 5-hydroxymethylfurfural in water. Green Chem..

[25] Schlesinger H.I., Brown H.C., Finholt A.E., Gilbreath J.R., Hoekstra H.R., Hyde E.K. (1953). Sodium borohydride, its hydrolysis and its use as a reducing agent and in the generation of hydrogen. J. Am. Chem. Soc..

[26] Balbay A., Saka C. (2018). Effect of phosphoric acid addition on the hydrogen production from hydrolysis of NaBH_4_ with Cu based catalyst. Energy Sources Part A Recovery Util. Environ. Eff..

[27] Xia Y., Pei Y., Wang Y., Li F., Li Q. (2023). Effects of various metal doping on the structure and catalytic activity of CoB catalyst in hydrogen production from NaBH4 hydrolysis. Fuel.

[28] Song J., Li R., Dong H. (2023). Controllable hydrogen production from NaBH_4_ hydrolysis promoted by acetic acid. Int. J. Hydrogen Energy.

[29] Musser M.T. (2005). Adipic acid. Ullmann’s Encyclopedia of Industrial Chemistry.

[30] Patel D.M., Gujarati V.P., Sumesh C.K., Pataniya P.M. (2024). Enhanced hydrolysis of NaBH_4_ using cobalt sulphide for hydrogen production. Inorg. Chem. Commun..

[31] Bohnet M. (2003). Ullmann’s Encyclopedia of Industrial Chemistry.

[32] National Center for Biotechnology Information (2023). PubChem Compound Summary for CID 196, Adipic Acid. https://pubchem.ncbi.nlm.nih.gov/compound/Adipic-Acid.

[33] Kennedy G.L. (2002). Toxicity of adipic acid. Drug Chem. Toxicol..

[34] Balbay A., Saka C. (2018). The effect of the concentration of hydrochloric acid and acetic acid aqueous solution for fast hydrogen production from methanol solution of NaBH_4_. Int. J. Hydrogen Energy.

[35] Ke D., Tao Y., Li Y., Zhao X., Zhang L., Wang J., Han S. (2015). Kinetics study on hydrolytic dehydrogenation of alkaline sodium borohydride catalyzed by Mo-modified Co–B nanoparticles. Int. J. Hydrogen Energy.

[36] Wang F., Luo Y., Wang Y., Zhu H. (2019). The preparation and performance of a novel spherical spider web-like structure Ru-Ni/Ni foam catalyst for NaBH_4_ methanolysis. Int. J. Hydrogen Energy.

[37] Kotkondawar A.V., Rayalu S. (2020). Enhanced H_2_ production from dehydrogenation of sodium borohydride over the ternary Co_0.97_Pt_0.03_/CeO_*x*_ nanocomposite grown on CGO catalytic support. RSC Adv..

[38] Wang F., Zhang Y., Luo Y., Wang Y., Zhu H. (2020). Preparation of dandelion-like Co–Mo–P/CNTs-Ni foam catalyst and its performance in hydrogen production by alcoholysis of sodium borohydride. Int. J. Hydrogen Energy.

[39] Guo J., Hou Y., Li B., Liu Y. (2018). Novel Ni–Co–B hollow nanospheres promote hydrogen generation from the hydrolysis of sodium borohydride. Int. J. Hydrogen Energy.

[40] Alshammari M., Alshammari K., Alhassan S., Alshammari A.H., Alotaibi T., Alotibi S., Ismael A., Taha T.A.M. (2024). A High-Performance Cr_2_O_3_/CaCO_3_ nanocomposite catalyst for rapid hydrogen generation from NaBH_4_. Nanomaterials.

